# User avoidance behavior in pharmaceutical e-commerce intelligent customer service: a stressor-strain-outcome perspective

**DOI:** 10.3389/fpsyg.2025.1514571

**Published:** 2025-02-04

**Authors:** Jing Jia, Lu Chen, Chengzhen Wu, Manling Xiao

**Affiliations:** ^1^School of Business, Changzhou University, Changzhou, China; ^2^School of Business, Hanyang University, Seoul, Republic of Korea; ^3^School of International Commerce, Konkuk University, Seoul, Republic of Korea

**Keywords:** intelligent customer service, pharmaceutical e-commerce, user avoidance behavior, cognitive overload, stressor-strain-outcome, electronic health literacy

## Abstract

**Introduction:**

This study explores the implementation of Intelligent Customer Service (ICS) in pharmaceutical e-commerce, aiming to enhance user acceptance and service efficiency while addressing the psychological factors influencing user behavior. It expands the boundaries of technology acceptance research by focusing on ICS use and avoidance in high-risk environments.

**Method:**

A total of 418 valid questionnaires were collected from participants, ensuring data quality through rigorous screening. The study employed SPSS for data normality tests and SmartPLS for structural equation modeling to analyze the relationships between emotional stress, system overload, and user avoidance behavior.

**Results:**

The findings indicate that system overload, information overload, and service overload significantly contribute to user emotional stress, which in turn drives avoidance behavior. The analysis revealed strong explanatory power (*R*^2^ values ranging from 0.450 to 0.586) and confirmed the mediating role of emotional stress in the relationship between overload factors and user avoidance.

**Discussion:**

This research highlights the critical role of emotional stress in user interactions with ICS, suggesting that pharmaceutical e-commerce companies must refine their ICS design to meet diverse user needs and reduce cognitive burdens. By leveraging big data and establishing robust feedback mechanisms, companies can enhance user experience and loyalty. The study also identifies limitations in demographic representation and suggests future research should incorporate qualitative methods for a deeper understanding of user behavior.

## 1 Introduction

As one of the most innovative technologies in the service sector, ICS is gradually reshaping the framework of marketing strategies and profoundly influencing consumer behavior (Davenport et al., 2020). Drawing on cutting-edge technologies such as natural language processing and machine learning, ICS enables efficient interaction with humans through text or voice. Industry and academia both recognize its capacity to enhance service capabilities and partially substitute traditional customer service models (Ciechanowski et al., 2019; Huang et al., 2021). Under the favorable policies of China’s pharmaceutical reform, several government departments have introduced a series of measures aimed at encouraging, guiding, and regulating the rapid development of the pharmaceutical e-commerce industry. As consumers increasingly embrace the convenience of online medicine purchases, 2023 has marked a significant growth wave in China’s pharmaceutical e-commerce sector, with the user base surpassing the 300 million mark (China Research and Intelligence Industry Institute, 2023). This escalating demand for consultations places substantial pressure on conventional customer service, prompting leading enterprises (Alibaba Health Pharmacy, JD Health, Meituan Medicine Purchase, and Dingdang Fast Medicine) to deploy ICS to optimize user experience, improve communication efficiency, and manage complex returns and complaints (Rai, 2020). The latest agency data indicates that China’s ICS market achieved a scale of 8.69 billion yuan in 2023 and is projected to further expand to 18.13 billion yuan by 2027, exhibiting an annual compound growth rate of 22.1% (China Commercial Industry Research Institute, 2023). This data underscores the robust development momentum of the ICS sector but also signals its vast future potential and unlimited possibilities. While the ICS market is experiencing significant growth, understanding the psychological mechanisms that lead to user avoidance is equally crucial for sustainable development.

Although prior evidence suggests that ICS significantly improves service efficiency and user experience, some pharmaceutical e-commerce platforms still report insufficient user stickiness and low continued usage. With the expansion of omnichannel shopping, higher demands are placed on ICS regarding interaction quality and user experience. Nonetheless, noticeable avoidance behaviors persist (Wu J. et al., 2023; Wu W. et al., 2023; Peng and Wu, 2024), which not only limit ICS integration but also create operational challenges and market pressure. In this context, it is crucial to examine why users sustain or avoid ICS after initial adoption, especially to clarify the underlying psychological mechanisms that affect user satisfaction, engagement, and the sustainable advancement of ICS. In response to these circumstances, this study does not concentrate on users’ initial decisions to adopt ICS; rather, it explores the drivers and processes that determine continued ICS usage or avoidance after preliminary acceptance. Existing literature has examined applications of ICS in finance, retail, and logistics, yet the psychological underpinnings of avoidance in highly sensitive pharmaceutical e-commerce settings remain insufficiently addressed (Zhan and Mao, 2024; Peng and Wu, 2024). Therefore, this research proposes following three research questions:

- RQ1: What theoretical framework underpins the analysis of the mechanisms influencing ICS avoidance behavior in pharmaceutical e-commerce?
- RQ2: What are the key factors that contribute to emotional stress during ICS interactions, and how do cognitive load and demands satisfaction influence this stress, ultimately leading to user avoidance?
- RQ3: How can governance measures be optimized to reduce ICS avoidance and enhance user engagement, specifically through strategies that improve demands satisfaction and increase e-health literacy?

Through comprehensive theoretical and empirical analyses, this study examines unmet user needs and underlying psychological processes, proposing strategies such as reducing cognitive load, reinforcing individualized needs satisfaction, and improving e-health literacy. These measures provide guidance for broader and deeper ICS implementation in pharmaceutical e-commerce, with the aim of increasing long-term user acceptance, boosting usage frequency, and ensuring the industry’s healthy, sustainable development. Major contributions include: (1) at the literature level, an expansion of technology acceptance and ICS research boundaries by highlighting ICS use and avoidance under high-risk, high-scrutiny conditions (pharmaceutical e-commerce); (2) at the theoretical level, the adoption of multiple perspectives—cognitive load, needs satisfaction, and e-health literacy—to deepen understanding of user emotions and behaviors in pharmaceutical e-commerce and establish an integrative framework for AI-driven customer service research; and (3) at the practical level, effective recommendations that address both enterprise and user needs, mitigating psychological pressure, improving interaction quality, and enhancing service efficiency. In sum, this study clarifies the core determinants and mechanisms of ICS avoidance in the pharmaceutical e-commerce context, offering valuable insights and references for subsequent academic inquiries and industrial applications.

## 2 Literature review

### 2.1 Intelligent customer service (ICS)

In recent years, scholars from various disciplines, including behavioral science, management, and marketing, have conducted extensive research on ICS users, yielding significant results. In the field of behavioral science, researchers have explored the drivers and barriers to the adoption and continued use of ICS from the perspective of technology diffusion (Luo et al., 2019; Wu et al., 2020; Ashfaq et al., 2020; Xie and Lei, 2022). These studies reveal the internal logic behind the diffusion of ICS technology and identify key factors affecting user acceptance. However, most studies primarily focus on financial, retail, or logistics contexts, while the highly regulated and potentially high-risk environment of healthcare e-commerce receives limited attention. Because personal health and medication safety are involved in this context, ICS users in healthcare e-commerce tend to exhibit greater caution and develop more complex demands and concerns, yet relevant research remains insufficient (Li et al., 2018). Large volumes of heterogeneous, multisource information often emerge alongside the rapid advancement of information and artificial intelligence technologies and frequently lead to cognitive overload and low utilization (Zhan and Mao, 2024; Peng and Wu, 2024). This situation easily provokes emotional stress, such as anxiety and frustration, which, in turn, cause users to avoid or reject technologies that are perceived as threatening. This phenomenon is especially evident in ICS, where only 20 to 30% of information is thoroughly read and effectively utilized (Wu J. et al., 2023; Wu W. et al., 2023). Although current studies provide valuable insights into satisfaction, loyalty, trust, and rejection (Munnukka et al., 2022; Hsu and Lin, 2023), deeper explorations into the mechanisms underlying ICS avoidance remain lacking. Meanwhile, ICS usage avoidance in healthcare e-commerce is rarely examined (Okuda and Shoda, 2018). Therefore, an in-depth investigation of ICS user avoidance behavior in this high-risk, high-concern environment not only refines the academic understanding of ICS user behavior but also provides more targeted implications for the healthy and sustainable development of the healthcare e-commerce industry.

### 2.2 Negative usage behavior

Currently, a unified and clear definition of negative usage behavior has not yet been established in academia. In the research conducted by various scholars, a range of non-continuous usage behaviors is innovatively categorized under the term “negative usage behavior.” This broad classification encompassed behaviors such as neglect and withdrawal, lurking, avoidance, information blocking, resistance, and knowledge hiding (Liu et al., 2017; Liu et al., 2018). Another research perspective suggests that negative behavior can be seen as users’ attempts to impede the use of information systems (Lapointe and Rivard, 2005), while some studies emphasize that negative usage behavior stems from the counteractive emotions triggered by changes in information systems (Kim, 2010). Through a systematic review of existing literature, it has been found that negative usage behavior among users in the Internet environment manifests in various forms. These include, but are not limited to, reduced usage frequency, temporary suspension or interruption of use, migration to alternative platforms, and eventual discontinuation. However, studies specifically examining this behavior within ICS usage on pharmaceutical e-commerce platforms remain limited, and the role played by emotional stress in triggering user avoidance has been insufficiently explored (Peng and Wu, 2024). Given the marked differences in user behavior across diverse online platforms, a precise delineation of the research context is crucial (Zhang and Gao, 2021). Current investigations predominantly focus on ICS adoption and continuous usage, centering on positive behaviors; by contrast, relatively little attention is devoted to avoidance behaviors precipitated by emotional distress. Furthermore, most research draws on the Theory of Rational Behavior, emphasizing cognitive judgments while rarely incorporating affective or emotional factors (Chen and Xiong, 2023). While some studies in the fields of information systems (IS) and consumer behavior have indeed begun to explore affective factors, such as the work by Qin et al. (2021a) and Qin et al. (2021b) on mobile augmented reality applications and their impact on continuous use and purchase intentions, the integration of these factors remains limited in the context of intelligent customer service (ICS) within healthcare e-commerce. For instance, Qin et al. (2021a) and Qin et al. (2021b) highlight the importance of a cognition-affect-conation perspective, and Bell et al. (2020) discuss motivational and affective factors influencing consumer behavior in e-commerce. However, these insights have not been sufficiently applied to understand user avoidance behavior in ICS, particularly in high-stakes environments like healthcare. Therefore, this research aims to enrich the research context of ICS interactions in pharmaceutical e-commerce by investigating the emotional dimensions that influence user interactions with ICS, thereby expanding the theoretical application boundaries and contributing to a more comprehensive understanding of user behavior in this critical area. In reality, emotions often act as a pivotal influence in information system use, at times critically shaping users’ beliefs, attitudes, and decisions (Ping, 2013). In view of this, this paper focuses on user avoidance behavior concerning ICS in pharmaceutical e-commerce. This behavior is defined as strategies such as avoidance, escape, inhibition, distraction, or control employed by individuals to reduce unpleasant experiences (Craske et al., 2008). As ICS gains traction on pharmaceutical e-commerce platforms and users’ service expectations continue to rise, emotional stress become more readily triggered, amplifying users’ inclination to reject or avoid ICS in favor of alternative services. Therefore, an in-depth investigation of the underlying mechanisms and influencing factors of such avoidance is of considerable significance for optimizing user experience and enhancing service efficiency.

### 2.3 Stressor-strain-outcome (S-S-O)

The S-S-O model, widely applied across disciplines such as psychology, sociology, and organizational behavior, provides profound insights into the psychological and behavioral evolution individuals undergo when confronted with stressors. Stressors are defined as threatening stimuli in the external environment, the variety and intensity of which significantly impact individuals. Strain is the dynamic adjustment process exhibited by individuals on physiological, psychological, and behavioral levels in response to these stressors, shaped by personal traits and coping strategies. Finally, the outcome reflects the individual’s comprehensive behavioral response following the experiences of stressors and strain (Koeske and Koeske, 1993). The S-S-O model’s structured analytical perspective offers strong theoretical support for investigating how various stressors intricately influence user behavioral responses (Ayyagari et al., 2011). In the context of using ICS on pharmaceutical e-commerce platforms, the S-S-O model provides a robust framework for analyzing user behavioral responses when confronted with specific stressors. The key elements of this model in this context are as follows: In pharmaceutical e-commerce, stressors may include information overload, insufficient technology trust, and the complexities of medical decision-making. These factors can lead users to feel discomfort and confusion when using ICS. When facing these stressors, users may experience physiological, psychological, and behavioral strains. For example, users might feel anxious, frustrated, or fatigued, and these emotional responses can significantly impact their decision-making processes and willingness to engage with the system. Ultimately, these strains may lead to user avoidance behavior, where individuals choose not to use the ICS anymore. Such avoidance not only affects users’ online shopping experiences but may also have negative implications for customer loyalty and business success within pharmaceutical e-commerce platforms.

Additionally, it is noteworthy that in a dynamic and ever-changing research contexts, the S-S-O model has proven to be a powerful tool for analyzing negative usage behaviors (Guo et al., 2020). For instance, some studies have used this framework to investigate the specific effects of pressure factors such as task complexity, task importance, and time urgency on the termination of online health information search (Yuan, 2021). Similarly, studies have developed a mechanism based on the S-S-O model that establishes a connection between excessive social media usage and individual academic performance, thereby expanding its application scope (Guo et al., 2020). Given the strongly negative characteristics inherent in user avoidance behavior, the S-S-O model is highly suitable for this context (Wang et al., 2019). The model’s explanatory power for negative usage behavior has been thoroughly validated across various fields. It not only exhibits robust explanatory capabilities in traditional areas like social networking and health information search but also successfully applies to emerging areas such as short videos, encompassing a range of behaviors like intermittent dropout, information overload, information fatigue, information avoidance, and usage avoidance (Yuan, 2021; Cao et al., 2018; Wang et al., 2019; Dhir et al., 2018; Ye et al., 2023; Duan et al., 2023). However, a review of existing literature indicates that research on avoidance of ICS in the high-sensitivity and high-risk context of pharmaceutical e-commerce remains insufficient. On one hand, most studies center on negative usage mechanisms in domains such as social media, online health service platforms, or short video applications, lacking sufficient discussion of stressors arising from information overload, limited technology trust, and complex medical decision-making—factors uniquely relevant to pharmaceutical e-commerce. On the other hand, although some scholars acknowledge the emotional stress (e.g., anxiety and frustration) that may be triggered during ICS usage, deeper explorations based on the S-S-O model to elucidate how these emotions lead to avoidance behavior are still not comprehensive (Wang et al., 2019). These observations suggest that there is ample room for further investigation, necessitating more empirical work to fully capture user avoidance of ICS in pharmaceutical e-commerce, as well as to provide targeted recommendations for platform or corporate improvements.

## 3 Research hypotheses and research model

### 3.1 Cognitive overload

Cognitive Load Theory, established by Australian psychologist Sweller in 1988, thoroughly analyzes how cognitive processing activities consume limited cognitive resources when individuals engage in learning and solving complex problems (Sweller, 1988). Once this consumption exceeds an individual’s working memory capacity, cognitive overload occurs (Zhang and Gao, 2021). Perceived overload, defined as an individual’s subjective experience when external information exceeds their processing capacity (Zha et al., 2020), has increasingly affected all internet users with the widespread adoption and development of the internet. Specifically, diverse online environments such as e-commerce, social networking interactions, and online information retrieval are closely linked users’ psychological experiences and perceptions of overload (Soto-Acosta et al., 2014; Bunjak et al., 2021). In the field of information systems research, perceived overload serves as a core concept widely described to reflect individuals’ subjective feelings when faced with cognitive overload. It can be further subdivided into various dimensions, including system overload, information overload, communication overload, service overload, social overload, and digital overload (Saegert, 1973; Yan et al., 2023). These classifications not only deepen the understanding of perceived overload phenomena but also lay a theoretical foundation for their application across different contexts. In examining the research on technology overuse, perceived overload is considered a critical antecedent to the negative effects of ICT usage (Karr-Wisniewski and Lu, 2010). Studies have shown that the overwhelming volume of online information juxtaposed with limited human cognitive capacity often results in physiological and psychological pressure, even provoking feelings of anxiety (Liu et al., 2017). In the context of ICS within pharmaceutical e-commerce, these overload phenomena can exacerbate users’ cognitive burdens and potentially lead to emotional exhaustion, manifested as negative emotional responses. Therefore, this paper focuses on the user group of ICS in pharmaceutical e-commerce and explores their avoidance behavior during usage. Grounded in the SSO model and integrated with Cognitive Load Theory, this study operationalizes stressors into three dimensions: system overload, information overload, and service overload. This refinement not only accurately identifies the key factors influencing user behavior but also establishes a robust theoretical basis for developing effective intervention strategies.

#### 3.1.1 System overload

When the complexity and functionality of information technology exceed an individual user’s effective processing capacity or when the features provided by the system far exceed the user’s actual needs, a phenomenon known as feature overload occurs, potentially leading to unnecessary psychological stress for users (Zhang et al., 2016). System overload specifically refers to the failure of information technology to enhance user efficiency and improve the user experience due to functional redundancy or usability flaws in design. Previous studies have extensively explored the relationship between system feature overload and user emotional states. Users often experience emotional stress as they continuously strive to adapt to the increasingly complex features of a system (Liu, 2022). Furthermore, research indicates that the intensifying complexity of system features exacerbates users’ cognitive load directly contributes to the emergence of emotional stress (Wang et al., 2022; Sheng et al., 2023). Qualitative studies suggest that while rapid advancements in digital technologies provide convenience to users, they also increase technological burdens—particularly when the usability of information technology tools is inadequate (Zhan and Mao, 2024). This lack of usability not only fails to enhance work efficiency but may also trigger negative emotional experiences for users. In the specific context of pharmaceutical e-commerce, users often find themselves in a state of urgency due to their need for medical consultations or drug information. If users encounter issues related to system overload—such as excessive complexity, slow response times, or inadequate intelligence—during interactions with ICS systems, they may experience negative emotional stress, including impatience, confusion, and dissatisfaction. Based on these insights, this study proposes the following hypothesis:

*H1*: System overload positively influences users’ emotional stress.

#### 3.1.2 Information overload

Information overload describes the dilemma individuals face when confronted with information demands that far exceed their processing capabilities (Eppler and Mengis, 2008). With the rapid development of the Internet, the volume of information has grown explosively, and the speed of updates continues to accelerate. Although information is intended to reduce uncertainty, an excess of information and specific types of data can have the opposite effect, increasing uncertainty and cognitive load, thereby inducing information overload (Huang et al., 2024). When individuals are inundated with information and are unable to effectively integrate, absorb, and utilize it, not only is information processing efficiency diminished, but it may also negatively impact their emotional states and decision-making processes (Shi et al., 2020; Sedera et al., 2022). Specifically, recent research suggests that the presence of excessive information can indirectly impede users’ intention to continue utilizing a system by intensifying psychological pressure and burdens through the mediating mechanism of cognitive inhibition (Yang and Kim, 2013). This phenomenon is particularly pronounced in the realm of health information. The findings of certain scholars indicate that individuals who perceive elevated levels of information overload frequently encounter heightened stress related to their health (Misra and Stokols, 2012). Translating this perspective to the pharmaceutical e-commerce environment, users engaging with ICS systems must perform a series of complex cognitive processing activities. When the volume of information provided by the ICS exceeds the user’s processing capabilities, it directly results in difficulties in understanding and processing information, which can trigger negative emotional responses, including anxiety, frustration, and anger (Li et al., 2018). It is particularly noteworthy that users of pharmaceutical e-commerce platforms often have relatively fragile physical or psychological conditions. In this context, information overload not only interferes with users’ effective filtering and identification of information but also exacerbates their usage pressure, ultimately potentially leading to heightened emotional stress. Therefore, based on these observations, this study proposes the following hypothesis:

*H2*: Information overload positively influences users’ emotional stress.

#### 3.1.3 Service overload

Service overload manifests primarily in forms such as delayed service responses, poor service quality, and the forced provision of unwarranted services (Zhang and Gao, 2021). In the specific context of pharmaceutical e-commerce, users maintain a highly cautious attitude towards the medical information provided by ICS. However, factors such as technological limitations, the complexity of inquiries, backend system load, and network delays contribute to delays in ICS responses. Such delays disrupt the expected interaction flow between users and the service system. When users experience waiting times for information or assistance, their cognitive resources are strained, leading to increased frustration and anxiety—both characteristics of emotional stress. Moreover, perceived inefficiencies in service can further exacerbate negative emotional states, potentially resulting in avoidance behaviors. Therefore, addressing service overload, including response delays, is crucial for enhancing user satisfaction and engagement within ICS (Wu J. et al., 2023; Wu W. et al., 2023). Additionally, frequent promotional services, advertising push notifications, and excessive content may disrupt normal cognitive and usage processes, heightening user concerns and increasing cognitive load. According to cognitive load theory, when service overload conflicts with users’ limited cognitive resources, it negatively impacts their emotional state, potentially triggering undesirable emotions such as anxiety and irritability (Peng and Wu, 2024).

In the current context, the pharmaceutical e-commerce industry in China is experiencing rapid growth, with the user base surpassing 300 million. This surge in users leads to a significant increase in consultation requests received by ICS systems, placing considerable pressure on system resources. At the same time, the limitations of intelligent technology in practical applications become increasingly apparent. Furthermore, the majority of e-commerce platforms have adopted ICS as the primary mode of service delivery. This “default” setting often compels users to engage with ICS involuntarily, which undoubtedly exacerbates users’ negative attitudes and stress responses. Based on this thorough analysis and discussion, this study proposes the following hypothesis:

*H3*: Service overload positively influences users’ emotional stress.

### 3.2 Emotional stress

Emotional stress represents an unconscious and aversive psychological response to stressful situations, encompassing a complex array of negative emotional experiences, including anxiety, irritability, and disappointment. The rapid advancement of information technology and the explosive increase in information volume have made emotional stress increasingly common and significant in online environments. When internet users encounter overly complex and diverse system functionalities alongside vast quantities of information, they often experience a sense of discomfort with the information technology environment, which subsequently increases their stress levels (Ragu and Tarafdar, 2008). This observation aligns with the concept of “technostress,” which views modern adaptation disorders caused by computer technology as a form of illness, negatively impacting individuals’ attitudes, thoughts, behaviors, and psychological states (Brod, 1984). The person-environment fit model provides a theoretical framework for understanding the origins of stress, emphasizing the importance of maintaining a balanced relationship between individuals and their surrounding environment (Cooper, 2001). When this balance is disrupted, stress arises, leading to corresponding strain responses. Additionally, research indicates that anxiety, as a specific manifestation of stress, often drives users to adopt strategies to avoid or reduce their frequency of internet use, resulting in avoidance behavior (Bozionelos, 2004). Additionally, studies reveal the critical role of emotional stress in driving individuals to engage in avoidance behaviors (Liu et al., 2017; Wang et al., 2018). Recent research indicates that emotional stress not only lead to avoidance behavior but may also result in users shifting to alternative services or entirely abandoning certain systems (Wang et al., 2022; Feng et al., 2022). Based on the cumulative analysis of the aforementioned theoretical background and empirical studies, it is proposed that within the pharmaceutical e-commerce context, users who experience negative psychological reactions, such as increased emotional stress, during interactions with ICS systems are likely to adopt coping strategies. These strategies may include reducing the frequency of use, ignoring customer service information, or even avoiding the use of ICS systems altogether.

Cognitive overload is often regarded as a significant external factor that triggers negative psychological reactions and behavioral intentions. Research reveals a significant positive association between perceived information overload and three negative psychological states—emotional stress, depression, and anger—experienced by online health information seekers, emphasizing the profound impact of this overload phenomenon on users’ psychological well-being (Swar et al., 2017). Similarly, it has been noted that continuous exposure to overload depletes individuals’ psychological resources, leading to intentions of discontinuous use, further corroborating the negative influence of overload on behavior (Maier et al., 2015). In the specific context of pharmaceutical e-commerce, the S-S-O model is employed to conceptualize overload as an external stressor (Stimulus) that impacts users of ICS systems, consequently triggering internal emotional stress (Stress) and ultimately leading to specific behavioral outcomes (Outcome). This logical chain clearly illustrates how the phenomenon of cognitive overload indirectly influences users’ behavioral intentions through emotional stress as a mediating variable. Therefore, emotional stress plays a crucial role as a mediator in the study of discontinuous use behaviors resulting from cognitive overload. For instance, research investigates how three types of overload—information, communication, and social—affect the internal psychological states, such as fatigue and regret, of social media users, ultimately prompting them to exit the platform (Cao and Sun, 2018). Additionally, studies highlight the role of emotional stress as a mediating variable between overload and information avoidance behaviors within the context of personal information management, further enriching the theoretical framework of this field (Zhan and Mao, 2024). Based on the analysis above, the following hypothesis is proposed:

*H4*: Users’ emotional stress positively influences their avoidance behavior.

*H5*: Users’ emotional stress acts as a mediating variable between cognitive overload (including system overload, information overload, and service overload) and avoidance behavior.

### 3.3 Demand satisfaction

Demand Satisfaction is defined as the extent to which an ICS effectively meets users’ diverse needs, including technical, informational, and psychological dimensions. The Uses and Gratifications Theory serves as a foundational framework in communication studies, emphasizing the audience’s perspective and explaining the motivations behind individuals’ active pursuit of media contact to fulfill specific needs (Zhang et al., 2023). In the context of AI-driven chatbot experiences, research highlights key attributes of this theory, such as information needs, emotional needs, and social interaction needs, all of which significantly enhance user experience. With rapid advancements in digital technology, the field of human-computer interaction is experiencing unprecedented opportunities for development (Cheng and Jiang, 2020). Studies indicate that AI-driven virtual assistants on smartphone platforms in Nigeria significantly increase user acceptance and satisfaction by effectively addressing diverse user needs. This trend underscores the importance of fulfilling user needs in human-computer interaction (Iyinolakan, 2023). However, the lack of need fulfillment can lead to negative emotional outcomes. Research warns that when technology fails to satisfy users’ psychological needs for escapism or social interaction, emotional stress such as anxiety may arise (Elhai et al., 2016). Similarly, unmet expectations for social gratification can also result in negative emotional responses (Thorisdottir et al., 2019). These findings collectively emphasize the central role of need fulfillment in shaping user experiences.

Given this research background, it is particularly necessary to consider user need fulfillment when examining negative usage behaviors in the context of ICS in pharmaceutical e-commerce. Specifically, the degree of need fulfillment refers to how effectively the ICS system and its functional services can meet users’ actual usage needs. This metric is significant for understanding the motivations and mechanisms underlying user behavior. User behavior is often guided by internal needs, prompting individuals to actively seek and utilize relevant services. In this process, the degree of need fulfillment becomes a core criterion for users to evaluate their service experience (Wu et al., 2022). In the specific context of pharmaceutical e-commerce ICS, service providers must not only focus on meeting users’ basic information needs regarding medication inquiries and disease knowledge but also enhance the convenience of system operation, the depth of emotional communication, and the richness of social interactions to comprehensively address users’ diverse needs.

Given these considerations, this study incorporates demand satisfaction as a key influencing factor into the research framework, closely aligned with the operational environment of pharmaceutical e-commerce. It systematically measures users’ need fulfillment during interactions with ICS from three dimensions: technical needs, information needs, and psychological needs. This multidimensional assessment approach aims to reveal the complexity of user needs and its multifaceted impact on service experience. Furthermore, when users perceive that the services provided by ICS fail to adequately meet their personalized needs in technical, informational, or psychological dimensions, this state of dissatisfaction may trigger negative psychological cognitions and emotional experiences. Consequently, it may lead users to adopt avoidance behaviors, such as reducing usage frequency, choosing alternative channels, or completely abandoning the use of the ICS system (Peng and Wu, 2024). The formulation of this hypothesis not only provides a new perspective for understanding users’ negative usage behaviors but also offers insights for optimizing ICS in pharmaceutical e-commerce to enhance user satisfaction. Based on the analysis above, the following hypotheses are proposed:

*H6*: Users’ demand satisfaction negatively affects their emotional stress.

*H7*: Users’ demand satisfaction negatively affects their avoidance behavior.

### 3.4 Electronic health literacy

In the current wave of digital transformation, e-health literacy has emerged as an indispensable core competency for citizens (Chen et al., 2024). This concept, initially introduced and further refined through multiple iterations, effectively integrates various dimensions, including traditional literacy, information literacy, media literacy, health literacy, information technology literacy, and scientific literacy. It precisely captures an individual’s comprehensive ability to efficiently acquire, deeply understand, accurately evaluate, and appropriately apply health information within a digital context (Norman and Skinner, 2006). With the rapid advancement of artificial intelligence technologies, research has foresightedly proposed the concept of eHealth literacy 2.0, emphasizing the importance of users’ interactive skills in online environments and their ability to effectively apply health information in practical situations (Van Der Vaart and Drossaert, 2017). Studies have robustly validated the close relationship between e-health literacy and users’ attitudes and behaviors in the context of health applications (Wu and Zhang, 2022; Zhang et al., 2023). As an important factor influencing individuals’ psychological states and behavioral choices, stressors can be categorized into two types: challenge stressors and hindrance stressors. The former is often viewed as a beneficial factor that promotes growth when adequately addressed, while the latter is widely perceived as negative stress due to its potential harmful effects (Podsakoff et al., 2007; Srivastava et al., 2015). Given that the nature of technological stress depends on the personal abilities and characteristics of the evaluator, this paper specifically considers eHealth literacy as an inherent characteristic of users while conducting an in-depth analysis of avoidance behavior in ICS within the pharmaceutical e-commerce environment, assigning it the role of a moderating variable. Research indicates that health information literacy plays a critical role in moderating exposure to misinformation and perceived threats, demonstrating that individuals with low health information literacy are more likely to experience anxiety and panic (Montagni et al., 2021; Pan et al., 2022). Furthermore, it has been revealed that e-health literacy, as a moderating variable, significantly affects user satisfaction and continuous usage intentions in online health communities (Fang, 2020). Based on the aforementioned theoretical foundations and empirical findings, the following hypothesis is proposed:

*H8*: Users’ e-health literacy moderates the relationship between cognitive overload (including system overload, information overload, and service overload) and emotional stress.

**Figure 1:**
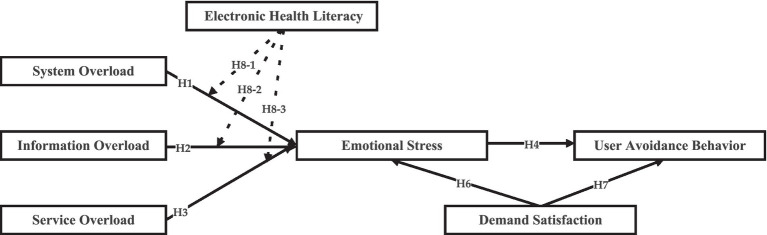
Theoretical model.

Based on the above hypothesis, a conceptual framework is constructed to examine avoidance behavior among users of ICS in pharmaceutical e-commerce, as illustrated in Figure 1.

## 4 Research methodology

### 4.1 Scale development and design

The questionnaire designed for this study features a rigorous structure divided into two main sections: basic information collection and primary scale assessment. The basic information section aims to gather key demographic details about respondents, including gender, age, educational background, and occupational type. The primary scale section is meticulously constructed to measure the relationships between key elements in the theoretical model and user avoidance behavior. To ensure the reliability of the research, the scales used in the questionnaire are adapted from established validated measures in the relevant fields. Additionally, they are carefully localized to align with the practical application of ICS within the pharmaceutical e-commerce context, ensuring their applicability and accuracy. The measurements for perceived system overload, perceived information overload, and perceived service overload are adapted from established scales in existing research, with four items developed for each construct (Saegert, 1973; Sheng et al., 2023; Huang et al., 2024; Sedera et al., 2022; Yang and Kim, 2013). Similarly, emotional stress was measured using four items (Zhan and Mao, 2024; Liu et al., 2017; Liu et al., 2018). User avoidance behavior (Peng and Wu, 2024; Liu et al., 2018), demand satisfaction (Guo et al., 2020; Zhang et al., 2023), and e-health literacy were each also measured with four items (Norman and Skinner, 2006; Fang, 2020). In terms of scale design, a five-point Likert scale is uniformly employed, ranging from 1 to 5. This format facilitates clear expression of attitudes and opinions by respondents. To further ensure the reliability and validity of the questionnaire, five experts in the field were invited to conduct a pre-test. Based on their valuable feedback, detailed adjustments and optimizations were made to the questionnaire, resulting in the finalized version presented in Table 1. This process not only enhances the scientific rigor of the questionnaire but also lays a solid foundation for subsequent data collection and analysis.

**Table 1:** Questionnaire items.

Construct	Item
System overload	The ICS has redundant functions. The operation of ICS is complex, making it difficult to quickly obtain medical information. The intelligence level of ICS is not high enough, and it cannot accurately understand my needs. ICS lacks humanization, and it feels less personal.
Information overload	The excessive information pushed by the ICS hampers concentration. The plethora of options recommended by the ICS leads to decision-making difficulties. The medical information provided by the ICS lacks precision. The information provided by the ICS is verbose and irrelevant, failing to meet academic journal standards in management studies.
Service overload	The response speed of ICS is slow. The quality of service provided by ICS is not high. The communication efficiency of ICS is low. ICS lacks emotional value.
Emotional stress	I feel anxious due to the lack of timely and effective assistance from ICS. I experience frustration or dismay when I am dissatisfied with ICS. I am fatigued and reluctant to continue engaging with ICS. I harbor a sense of aversion towards the services provided by ICS, believing that they fail to meet my needs.
User avoidance behavior	I will reduce my reliance on AI customer service. I will actively seek to avoid the information conveyed by AI customer service. I will pursue assistance through alternative channels, such as human customer service or telephone consultations. I will refrain from utilizing certain functions of AI customer service or transition to other platforms.
Demand satisfaction	ICS offers high convenience for medical consultation and pharmaceutical purchases. The accuracy of medical information and advice provided by ICS is noteworthy. ICS effectively meets my informational needs regarding medical and pharmaceutical knowledge. Interacting with ICS provides a sense of emotional support and understanding that is sufficient for my needs.
eHealth literacy	I am confident in utilizing ICS. I am knowledgeable about how to consult ICS to obtain the necessary medical services. I can comprehend the medical advice and instructions provided by ICS. I can assess whether the medical information provided by ICS is accurate.

### 4.2 Data sources and data characteristics

This study focuses on users with experience in using ICS systems in the field of pharmaceutical e-commerce. Recruitment of participants was primarily facilitated through prominent pharmaceutical e-commerce platforms in China, including AliHealth, JD Health, Meituan Buying Medicine, and Dingdang Kuaiyao. These platforms are distinguished by their substantial brand recognition, extensive user base, and significant market shares. Data collection occurred between May and July 2024, utilizing a hybrid approach of online and offline methodologies. The online component involved the dissemination of a questionnaire link via the “Wenjuanxing” platform, supported by electronic invitations and promotion through online communities and social media. To incentivize participation, a series of electronic digital rewards were provided to users who completed the online survey. The offline component consisted of distributing printed questionnaires to individuals with ICS experience at pharmacies, community health service centers, and universities within the Yangtze River Delta region. Participants were also offered small gifts of comparable value upon completion of the paper questionnaires. The recruitment and screening criteria stipulated that participants must be at least 18 years of age and possess fundamental online purchasing competencies. Questionnaires completed in fewer than 5 min or exhibiting a pattern of repetitive answers were classified as invalid. In total, 483 questionnaires were collected, and following rigorous scrutiny, 418 valid responses were retained, yielding an effective response rate of 86.58%. This sample exhibits strong representativeness regarding geographical distribution and ICS usage experience, thereby providing a robust foundation for subsequent data analysis.

The latest research focuses on the benchmark company in China’s pharmaceutical e-commerce industry, 111 Group, providing an in-depth analysis of user profiles (iiMedia Research, 2023). The results indicate that the user base of pharmaceutical e-commerce platforms continues to expand, with female users dominating at 67.4%, while male users account for 32.6%. In terms of age demographics, the young adult group (ages 25–35) is particularly prominent, representing 60.4% of users. This finding aligns with the demographic analysis results presented in this paper. A detailed examination of demographic characteristics reveals specific outcomes, as shown in Table 2. In terms of gender composition, female respondents also represent a significant proportion, totaling 237 individuals, or 56.70% of the sample. This predominance may be attributed to the traditional role of women as key decision-makers in household shopping within Chinese culture. Regarding age distribution, the majority of respondents are aged between 26 and 45, totaling 289 individuals and accounting for 69.1%. This reflects the rapid adoption of emerging technologies and the deep integration of digital lifestyles among the younger generation. In terms of educational attainment, respondents with a bachelor’s degree constitute the highest proportion, totaling 208 individuals or 49.76%. This not only demonstrates the achievements of China’s higher education popularization but also suggests that individuals with higher education levels tend to utilize e-commerce platforms for pharmaceutical purchases. As for occupational distribution, employees from enterprises, government, and public institutions form the majority, accounting for 75.60%, or 316 individuals. This trend may result from the time constraints faced by professionals, who are more likely to prefer convenient and efficient e-commerce channels for their daily shopping needs, including the purchase of pharmaceutical products.

**Table 2:** Sample’s demographic profile.

Measure	Items	Frequency	Percent
Gender	Male	181	43.30
	Female	237	56.70
Age	≤25 years old	70	16.75
	26 ~ 35 years old	120	28.70
	36 ~ 45 years old	169	40.40
	≥46 years old	59	14.10
Education level	High School and Below	98	23.44
	Bachelors	208	49.76
	Masters and above	112	26.79
Occupation	Employees of an enterprise	118	28.23
	Civil Servant	198	47.37
	Retirement	73	17.46
	Unemployed	29	6.94
	Other	66	17.8

### 4.3 Data normality test and common method variation test

To ensure that the sample data used in this study is both accurate and scientifically valid, the primary step is to verify whether these data follow a normal distribution. This measure minimize potential biases in the results of structural equation modeling hypotheses arising from non-normally distributed data. The study employs SPSS 25.0 to conduct a thorough examination of skewness and kurtosis for the sample data obtained through a questionnaire survey. The results indicate that the absolute skewness coefficients range from 0.011 to 1.197, all of which do not exceed the threshold of 2. Similarly, the absolute kurtosis coefficients range from 0.026 to 1.325, also falling below the limit of 2. These two metrics collectively confirm that the sample data meet the criteria for normal distribution, thus establishing a solid data foundation for subsequent hypothesis testing using SEM. Additionally, given that the data in this study are sourced from an online questionnaire platform and collected via self-reporting methods, there exists a potential risk of common method variance that could threaten the validity of the research conclusions. To effectively address this challenge, several preventive measures were implemented during the data collection phase, including the administration of anonymous surveys, careful selection of questionnaire items, and the incorporation of reverse-scored items, all aimed at minimizing common method bias. To further verify the actual impact of common method variance in the sample data, an in-depth analysis using Harman’s single-factor test was conducted. Factor analysis was performed on all the questionnaire items using SPSS 25.0, revealing that seven factors with eigenvalues greater than one were extracted in the unrotated exploratory factor analysis. Notably, the variance explained by the largest factor was only 32.72%, well below the 50% criterion. This finding solidly demonstrates the absence of significant common method variance issues in this study, thereby enhancing the reliability and validity of the research outcomes.

## 5 Data analysis and research findings

### 5.1 Model validation

To verify the reliability and validity of the measurement model, a comprehensive statistical analysis of the latent variables and their corresponding observed variables is conducted, including key indicators such as Cronbach’s *α* coefficient, standardized factor loadings, composite reliability (CR), average variance extracted (AVE), and the square root of AVE, as detailed in Table 3. Regarding reliability, the data analysis reveals that all variables in this study exhibit Cronbach’s α coefficients significantly exceeding the commonly accepted threshold of 0.7 (Nunnally and Bernstein, 1994). Additionally, the CR values for all variables also surpass the 0.7 standard, indicating strong internal consistency among the measurement items, thus confirming the high reliability of the measurement model (Fornell and Larcker, 1981). In terms of validity, the factor loadings for all observed variables exceed the benchmark of 0.6 (Hair et al., 2010). Furthermore, the AVE for each variable meets the threshold of 0.5 (Fornell and Larcker, 1981). Considering these reliability and validity assessment indicators, it can be asserted that the measurement model of this study adheres to the stringent standards of convergent validity.

**Table 3:** Reliability analysis of scale content.

Constructs	Indicators	Loadings	a	CR	AVE
System overload	STO 1	0.881	0.820	0.895	0.740
	STO 2	0.845			
	STO 4	0.855			
Information overload	IMO 1	0.912	0.815	0.901	0.820
	IMO 2	0.922			
Service overload	SVO 1	0.866	0.824	0.898	0.747
	SVO 2	0.862			
	SVO 4	0.865			
Emotional stress	NE 1	0.851	0.802	0.880	0.711
	NE 2	0.834			
	NE 3	0.844			
User avoidance behavior	UAB 1	0.854	0.821	0.847	0.734
	UAB 2	0.873			
	UAB4	0.860			
Demand satisfaction	DS1	0.867	0.852	0.877	0.782
	DS 2	0.874			
	DS 4	0.901			
eHealth literacy	eHL 1	0.899	0.856	0.897	0.813
	eHL 2	0.853			
	eHL 4	0.904			

a, Cronbach’s alpha; CR, composite reliability; AVE, average variance extracted.

To ensure the validity of the research findings, this study also conducted a non-response bias test and HTMT (Heterotrait-Monotrait Ratio) analysis to verify discriminant validity. To assess the potential impact of non-response bias, we compared the means of key variables (e.g., emotional stress, system overload) between early responders and late responders. An independent samples t-test revealed no significant differences between the two groups (*p* > 0.05), indicating that the impact of non-response bias is minimal. Moreover, to further validate the discriminant validity of the measurement model, we computed the HTMT values, which quantify the ratio of inter-construct correlations to intra-construct correlations (Henseler et al., 2015). According to Henseler et al. (2015), HTMT values should be below 0.85. Our analysis showed that all constructs had HTMT values below 0.85, corroborating strong discriminant validity among the constructs. Additionally, when the square root of each variable’s AVE is greater than its correlation coefficients with all other variables, the scale is considered to possess high discriminant validity (Fornell and Larcker, 1981). Additionally, all items show a variance inflation factor (VIF) of less than 5, indicating the absence of multicollinearity issues (Hair et al., 2010). Detailed results are presented in Table 4.

**Table 4:** Correlation matrix and discriminant validity.

Construct	STO	IO	SVO	NE	UAB	DS	eHL
STO	*0.860*	0.742	0.823	0.800	0.780	0.635	0.770
IO	0.671	*0.905*	0.782	0.753	0.720	0.810	0.771
SVO	0.727	0.667	*0.864*	0.816	0.798	0.669	0.580
ES	0.718	0.669	0.715	*0.843*	0.698	0.670	0.811
UAB	0.702	0.645	0.662	0.586	*0.859*	0.728	0.690
DS	0.534	0.702	0.549	0.541	0.608	*0.884*	0.531
eHL	0.684	0.665	0.481	0.696	0.583	0.531	*0.902*

Diagonal elements in italics are the square roots of AVE; Lower-triangle off-diagonal elements are correlation coefficients; Upper-triangle values in parentheses are HTMT values; All HTMT values are below the recommended threshold of 0.85, supporting discriminant validity.

### 5.2 Hypothesis verification

#### 5.2.1 Direct effect testing

This study employs SmartPLS 3.0 statistical analysis software, utilizing partial least squares (PLS) method to examine the data. In the PLS analysis, the evaluation of the hypothetical model includes path coefficient estimation and *R*^2^ values. The path coefficients indicate the direction and magnitude of influences among latent variables. The *R*^2^ value reflects the extent to which endogenous latent variables can be explained by exogenous latent variables, thereby showcasing the explanatory power of the model. The analyses yield *R*^2^ values of 0.486, 0.586, and 0.450 for emotional stress, user avoidance behavior, and need fulfillment, respectively, which are indicative of good explanatory capability. Additionally, the corresponding *Q*^2^ values are all greater than 0, confirming strong predictive capability. The results of the path coefficients and hypothesis testing are presented in Table 5. The findings reveal that all direct relationships in the model receive support. The standardized path coefficients for system overload, information overload, and service overload on emotional stress are 0.368, 0.362, and 0.172, respectively, all of which are significant, thereby supporting hypotheses H1, H2, and H3. The standardized coefficient for emotional stress on user avoidance behavior is 0.376, with empirical data supporting this influence, confirming hypothesis H4. Finally, the standardized path coefficients for need fulfillment on emotional stress and user avoidance behavior are −0.368 and −0.235, respectively, receiving empirical validation for hypotheses H6 and H7.

**Table 5:** Path coefficients and hypothesis test results.

Path	SPC	STDEV	*T*-statistic	*p*	Results
H1 STO(+) → ES	0.368	0.071	4.683	***	Supported
H2 IMO(+) → ES	0.362	0.069	4.464	***	Supported
H3 SVO(+) → ES	0.172	0.072	3.370	0.036*	Supported
H4 ES(+) → UAB	0.376	0.098	3.894	***	Supported
H6 DS(−) → ES	−0.368	0.058	3.554	***	Supported
H7 DS(−) → UAB	−0.235	0.073	3.085	0.002**	Supported

*p < 0.05; **p < 0.01; ***p < 0.001.

#### 5.2.2 Mediation effect test

To assess the mediating effects, the Bootstrap method is employed to examine the mediating role of emotional stress. A total of 5,000 bootstrap samples are generated to evaluate the mediating effect of emotional stress in the relationship between system overload, information overload, and service overload on user avoidance behavior, under the condition of a 95% confidence interval. The presence of the mediating effect is confirmed if the confidence interval does not include 0. The verification results, presented in Table 6, indicate the existence of an indirect effect, thus supporting hypothesis H5.

**Table 6:** Path coefficients and hypothesis test results.

Path	Effect-size	STDEV	Confidence Interval	Intermediate Effect
H5-1 STO → ES → UAB	0.061	0.028	(0.016, 0.127)	Supported
H5-2 IMO → ES → UAB	0.095	0.040	(0.056, 0.239)	Supported
H5-3 SVO → ES → UAB	0.065	0.039	(0.033, 0.186)	Supported

#### 5.2.3 Moderation effect test

As shown in Table 7, the study validates the role of e-health literacy as a moderating variable in the relationships between system overload, information overload, and emotional stress. To assess this moderating effect, a hierarchical regression analysis is employed. Specifically, a basic model is established that includes independent variables (system overload, information overload), the dependent variable (emotional stress), and control variables (such as age, gender, and education level). In the second model, the moderating variable (e-health literacy) is added to examine its impact on the relationship between the independent and dependent variables. The analysis is conducted using SPSS 26.0, and all variables are standardized to mitigate the effects of multicollinearity on the results. The findings indicate that e-health literacy significantly moderates the relationship between system overload and emotional stress (*β* = X, *p* < 0.05), supporting hypothesis H8-1. Additionally, e-health literacy significantly moderates the relationship between information overload and emotional stress (β = Y, p < 0.05), supporting hypothesis H8-2. However, e-health literacy does not significantly moderate the relationship between service overload and emotional stress (β = Z, *p* > 0.05), leading to the rejection of hypothesis H8-3.

**Table 7:** Moderating variable test.

Path	Correlation coefficient	*p*-value	Test results
H8-1 STO*eHL-ES	0.269	0.026	Supported
H8-2 IMO*eHL-ES	0.205	0.040	Supported
H8-3 SVO*eHL-ES	0.120	0.256	Not Supported

### 5.3 Research findings

This study employs rigorous empirical analysis to conclusively validate that system overload, information overload, and service overload significantly impact user emotional stress within the context of ICS in pharmaceutical e-commerce. These findings align with prior research, such as Zhang and Gao (2021) and Zhan and Mao (2024), which also emphasize the negative effects of information overload, system overload, and service overload on user anxiety and engagement in online health services and information storage contexts. The research further demonstrates that the cognitive load experienced by users and the emotional stress accumulated during interactions with ICS systems are key drivers behind their avoidance strategies. Despite the advantages of ICS in terms of convenience and personalized service experiences, users often encounter excessive product recommendations and irrelevant health information during their interactions. These issues complicate customer service solutions and compel users to exert additional effort in filtering and comprehending the information, ultimately weakening their information processing capabilities and increasing anxiety levels. This conclusion is consistent with findings from Peng and Wu (2024), who utilized grounded theory to explore avoidance behavior and identified negative emotions and emotional exhaustion as proximal outcomes leading to avoidance. Simultaneously, this study uniquely highlights emotional stress as a mediating factor triggering user avoidance behavior, a relationship that has not been fully explored in the existing literature. The explosive increase in information disrupts the balance between individuals and their surrounding environment, universally raising stress levels, a phenomenon widely acknowledged in academic circles (Brod, 1984; Cooper, 2001).

Moreover, user demand satisfaction significantly negatively affects emotional stress and avoidance behavior. The research by Peng and Wu (2024) reveals through interview results that when services fail to meet individual demands (technological, service, and psychological demands), users’ cognition and emotions are stimulated, leading to avoidance behavior. Conversely, in the ICS environment of pharmaceutical e-commerce, when users’ demands are quickly and accurately identified and effectively met, they experience positive feedback and feel understood and valued. This efficient interaction process reduces the frequency of interactions between users and ICS and shortens the time needed to obtain necessary information or services, thereby alleviating the cognitive load associated with interaction uncertainty and repetitiveness. Consequently, when users’ demands are accurately identified and effectively fulfilled, their satisfaction and loyalty towards ICS increase. Lastly, e-health literacy plays an important moderating role between cognitive overload and emotional stress. Existing studies indicate that users with higher e-health literacy exhibit stronger information processing and technological adaptation skills (Zhang et al., 2023). When utilizing ICS, their interactions are smoother, effectively reducing cognitive load and emotional stress caused by overload. Importantly, these users demonstrate greater confidence and decision-making abilities when faced with complex medical or health-related information, allowing them to utilize online resources more effectively to address health concerns. Therefore, the moderating effect of e-health literacy not only helps users maintain a positive and stable emotional state and promotes effective health management behaviors, but also enhances the user experience and service efficiency of ICS on pharmaceutical e-commerce platforms.

## 6 Conclusion

### 6.1 Theoretical contribution

This study conducts an in-depth analysis of user avoidance behavior in the context of ICS in pharmaceutical e-commerce, addressing the significant concerns surrounding user health and safety in this field. By constructing a systematic model, it comprehensively identifies and analyzes the key factors that drive users to avoid utilizing this service. The findings indicate that, despite the efforts of many pharmaceutical e-commerce companies to implement ICS to enhance service efficiency and user experience, the actual acceptance and usage rates among users fall short of expectations, particularly regarding the psychological factors influencing user behavior, which have not been adequately explored in the existing literature.

This research advances several specific theories, notably cognitive load theory, uses and gratifications theory, and e-Health literacy theory. By demonstrating how cognitive overload directly influences emotional stress and subsequent user avoidance behavior, the study extends the application of cognitive load theory to the high-stakes environment of pharmaceutical e-commerce. Additionally, it contributes to uses and gratifications theory by highlighting the importance of individualized needs satisfaction in enhancing user engagement with ICS, identifying specific user needs related to technology, information, and emotional support. Fulfilling these demands can significantly reduce avoidance behavior and increase user loyalty. Furthermore, the integration of e-health literacy theory emphasizes the role of users’ health literacy in moderating the effects of cognitive overload and emotional stress, illustrating that users with higher e-health literacy are better equipped to navigate complex information, thereby enhancing their overall experience with ICS and reducing the likelihood of avoidance.

This research not only provides new theoretical perspectives for understanding the complex psychological mechanisms involved in intelligent service processes but also significantly enriches the theoretical framework concerning user behavior in the domain of pharmaceutical e-commerce ICS. By integrating these theories, this study establishes a comprehensive theoretical framework that examines how users’ emotional states influence their avoidance behavior. Additionally, the research implements detailed localization adjustments to ensure that measurement tools are applicable and accurate within the context of pharmaceutical e-commerce in China, thereby laying a solid foundation for future related research. Finally, this study validates the mediating effect of emotional stress between overload and user avoidance behavior, effectively addressing the relative scarcity of discussions on the emotional dimension within current research on user behavior in information systems. This finding not only enhances the theoretical understanding of how user emotions influence technology usage behavior but also provides significant practical guidance for pharmaceutical e-commerce companies in optimizing ICS and improving user experience.

### 6.2 Practical insights

In the Chinese pharmaceutical e-commerce sector, the industry is experiencing unprecedented growth, driven by robust government reform policies. As the user base continues to expand and online medication purchasing habits mature, traditional manual customer service models face significant challenges in effectively addressing the surge in consultation demands. In this context, the introduction of ICS emerges as a necessary solution, providing pharmaceutical e-commerce companies with new avenues to manage service pressure. User experience and emotions play a crucial role in interactions with ICS; they are not only important drivers for upgrading the ICS industry but also key strategic considerations for pharmaceutical e-commerce companies aiming to accurately understand user needs and enhance market competitiveness. In light of this, this study focuses on empirical research regarding avoidance behavior from the user perspective and offers the following targeted recommendations.

First, ICS systems, leveraging the integration of advanced technologies such as natural language processing and machine learning, have become key drivers for enhancing service quality. However, with the widespread adoption of intelligent technologies and the increasing variety of alternative services in the market, users are setting stricter standards for the design and optimization of ICS systems. In light of this, pharmaceutical e-commerce companies need to focus on the refined design of ICS systems, aiming to meet diverse user needs while simplifying the complexity of system functions to ensure accurate and relevant information delivery. To achieve this goal, companies should strive to provide immediate and high-quality ICS to effectively alleviate service overload and reduce users’ cognitive burdens during information processing. By optimizing service processes and improving response times, pharmaceutical e-commerce companies can significantly enhance user efficiency, satisfaction, and loyalty. Furthermore, this efficient and considerate service experience helps reduce the occurrence of emotional stress among users, thereby further enhancing their overall experience.

Second, companies must further enhance their ability to meet user needs. In the specific context of ICS in pharmaceutical e-commerce, users often possess multi-layered and multidimensional requirements related to technology, information, and psychology. To accurately capture and fulfill these personalized needs, companies should leverage big data technologies to construct a comprehensive and precise user database. By deeply analyzing user behavior data, companies can gain a more nuanced understanding of user preferences and expectations, enabling them to provide ICS that aligns closely with user needs. This approach not only helps increase user acceptance of ICS but also significantly enhances users’ willingness to engage and their loyalty. Simultaneously, companies should establish a robust user feedback mechanism, which serves as an essential basis for service optimization and iteration. By regularly collecting, organizing, and analyzing user feedback, companies can promptly assess the effectiveness of the ICS and user satisfaction, thereby identifying areas for improvement. On this foundation, companies should swiftly adjust their service strategies and optimize service processes and content to better adapt to users’ evolving needs. This user-centric, continuously improving service model will aid companies in maintaining a competitive edge in a fiercely competitive market.

Finally, the level of individual e-health literacy among users becomes a crucial factor influencing the service experience and effectiveness when using ICS in pharmaceutical e-commerce. In this regard, pharmaceutical e-commerce companies need to implement a series of measures to enhance user experience and promote the widespread acceptance of ICS. On one hand, companies should focus on optimizing the presentation of information to ensure that it is both accurate and easily comprehensible. Specifically, the use of obscure technical jargon and lengthy explanations should be minimized, while employing visual aids, video tutorials, or concise textual descriptions in intuitive formats can help reduce users’ cognitive load, enabling them to quickly and accurately grasp key information points. On the other hand, companies should actively take on the responsibility of enhancing users’ e-health literacy. By developing online courses, creating interactive learning guides, and establishing a Q&A section on their websites, companies can offer diverse learning resources to users, guiding them toward acquiring essential medical knowledge and information retrieval skills. This proactive approach to user education not only fosters trust and reliance on ICS but also encourages users to more independently and effectively utilize the ICS platform for obtaining necessary medical information. In summary, by simultaneously optimizing information presentation and enhancing users’ e-health literacy, pharmaceutical e-commerce companies can significantly improve user experience and satisfaction with ICS, thereby driving broader acceptance and usage of this service and injecting strong momentum into the sustainable and healthy development of the industry.

## 7 Limitations and further research

First, regarding the demographic sample, the data in this study primarily focuses on individuals aged 26 to 45, with most participants holding a college degree or higher. This age group exhibits relatively mature digital habits and high levels of e-health literacy. However, this focus results in significant shortcomings in the balance and representativeness of the sample. Therefore, future research should adopt more scientific methods to comprehensively reflect diverse demographic characteristics, providing more valuable insights and recommendations for the field. Second, although the research method employed in this study is primarily based on rigorous quantitative analysis, it remains somewhat singular and uncertain. User avoidance behavior, as a prediction based on psychological changes, may overlook certain details of users’ internal activities when assessed solely through structured surveys that yield static cross-sectional data. To address this limitation, future studies could incorporate qualitative analysis methods, such as interviews, or utilize contextual experiments to provide a more comprehensive and nuanced understanding of respondents’ perceptions of the research subject. This approach would enhance the authenticity and objectivity of the research findings.

## Data Availability

The raw data supporting the conclusions of this article will be made available by the authors, without undue reservation.
